# The Dynamic Interplay of Affective, Cognitive and Contextual Resources on Children’s Creative Potential: The Modulatory Role of Trait Emotional Intelligence

**DOI:** 10.3390/jintelligence11010011

**Published:** 2023-01-04

**Authors:** Sergio Agnoli, Serena Mastria, Giacomo Mancini, Giovanni Emanuele Corazza, Laura Franchin, Tiziana Pozzoli

**Affiliations:** 1 Department of Life Sciences, University of Trieste, Via Weiss 2, 34128 Trieste, Italy; 2 Marconi Institute for Creativity, University of Bologna, Viale Risorgimento 2, 40136 Bologna, Italy; 3 Department of Psychology, University of Bologna, 40127 Bologna, Italy; 4 Department of Science Education, University of Bologna, 40126 Bologna, Italy; 5 Department of Electrical, Electronic, and Information Engineering “Guglielmo Marconi”, University of Bologna, 40136 Bologna, Italy; 6 Department of Psychology and Cognitive Sciences, University of Trento, 38122 Trento, Italy; 7 Department of Developmental and Social Psychology, University of Padua, 35131 Padua, Italy

**Keywords:** trait emotional intelligence, creative potential, executive functions, teachers’ beliefs about creativity, children, gender

## Abstract

In the present work we explored in two separate studies the modulatory role of trait emotional intelligence (EI) over the effect exerted on children’s creative potential by two other key elements defining creativity, namely cognitive resources (here explored through basic executive functions, Study 1) and contextual-environmental factors (that is, teachers’ implicit conceptions of the factors influencing children’s creativity, Study 2). Confirming previous research, executive functions (particularly interference control and working memory) emerged as main predictors of children’s creative performance; however, their positive effect arose especially when associated with a high trait EI level. In the same vein, teachers’ implicit conception about children’s creative potential and about their efficacy in teaching creativity emerged to exert a facilitatory effect on children’ creative potential. This effect occurred particularly when associated with low trait EI levels, affecting differently girls and boys. Trait EI emerged from these studies as an important individual resource to consider in order to understand the potential benefit of other (cognitive and contextual-environmental) resources on children’s creative potential. The implications on the role of trait EI as a constitutional element of children’s creativity, capable of promoting the expression of their creative potential, are discussed.

## 1. Introduction

It is widely accepted that creativity represents one of the most important 21st century skills, being constantly denoted as a main driver for the future of the current and of the next generations (Barbot et al. 2015; Corazza 2017; Davies et al. 2018; Glaveanu et al. 2020; Puccio 2017). More than ever before, there exists a pressing need, especially for educational purposes, to understand the elements required for the expression of individual creative potential, which is the latent ensemble of components that come at play in the production of potentially original and valuable ideas, i.e., creative ideas (Barbot et al. 2015, 2016; Lubart et al. 2013). Specifically, according to the multivariate approach (Lubart 1999; Sternberg and Lubart 1995), creative potential comes as a result of the confluence of several distinct, but interrelated resources. These resources for creativity are specific aspects of intelligence, knowledge, cognitive style, personality, motivation, affect, and physical and socio-cultural environmental contexts (Lubart et al. 2013; Sternberg and Lubart 1991) that converge in an interactive manner to define various creative abilities, which can be effective in realizing several creative projects and products that are then evaluated within specific sociocultural contexts. These resources are at the basis of creative potential and can be aggregated into three broad categories: cognitive-affective resources, conative resources^1^, and environmental-contextual resources (Sternberg and Lubart 1996). Cognitive, affective, and conative resources are person-centered factors for creativity, whereas environmental resources are context-centered factors. The importance of such resources for identifying creativity among schoolchildren is a matter of investigation, especially the way in which these components interactively converge, through summing or compensatory mechanisms. Here, we embrace this view, according to which creativity does not result from the sum of single components at an individual’s level, but rather from an interactive ensemble of resources involving person-centered and context-centered factors (Sternberg and Lubart 1995; Lubart et al. 2013). Specifically, the present work aimed at understanding how affective resources, conveyed by emotional intelligence, interacted with cognitive resources (Study 1) and with contextual resources (Study 2) in predicting children’s creative potential in a school environment. Cognitive resources were here explored through the measurement of basic executive functions (i.e., response inhibition, working memory, interference control) in children, since they are among the most explored cognitive constituents of the creative cognition (e.g., Beaty et al. 2014; Benedek and Fink 2019); whereas contextual resources were analyzed by considering the teachers’ implicit conceptions of the factors influencing children’s creativity, being that these conceptions are highly explored as contextual factors influencing the teaching of creativity and students’ creative potential as well (e.g., Gralewski and Karwowski 2019; Machts et al. 2016; Rubenstein et al. 2013).

### 1.1. The Relationship between Executive Functions and Creative Performance

The role of executive functions on creativity has been extensively explored in the prediction of creative behavior, defined in terms of objective performance and actual achievement, which are product oriented, especially in adult populations. The study of creative cognition, in particular, has traditionally focused on the association between executive functions and creative performance (Beaty et al. 2014; Benedek and Fink 2019; Benedek et al. 2014). For instance, several studies showed that creativity is associated with better response inhibition (Benedek et al. 2012; Zabelina et al. 2019), which is conventionally defined as the ability to interrupt or delay an action and to be able to reflect rather than display impulsive behavior. In the context of creative behavior research, the ability to produce a new idea is indeed related to the ability to inhibit and avoid common paths to generate unique and uncommon ideas (Benedek et al. 2014). Moreover, creative performance has been associated with the functioning of the working memory (WM), which involves holding information in mind and mentally working with it, and with cognitive flexibility, which is associated with the ability to change perspectives, showing that such abilities are useful skills to combine, in an original way, concepts that were previously not related (De Dreu et al. 2012; Mastria et al. 2021). People with higher working memory abilities indeed appear to be more able to overcome the interference exerted by automatic and non-original solutions. Research demonstrated how the relationship between executive functions and creativity is also evident in real-life contexts, showing, for instance, that high creative achievers (artists or design students as compared to non-artists or non-design students, respectively) have a higher ability to sustain attention for longer periods, revealing better cognitive control, which enables individuals to suppress attention and responses to irrelevant information in comparison to non-experts (Edl et al. 2014; Kozbelt 2008). Moreover, artists as compared to non-artists seem to be more able to switch flexibly towards more original ideas while showing a higher ability to regulate and control their thought and behaviors (see Zabelina et al. 2019). Overall, these results demonstrate that executive functions such as response inhibition and interference control as well as the performance of the working memory are basic cognitive underpinnings of the creative thinking process. Recently, research also focused on understanding the role of executive functions in creative performance during development (Krumm et al. 2018; Sampedro and Pena 2019; Stolte et al. 2020); however, much work remains to be done in this vein. In line with the results emerging with adults, executive functions such as flexibility and working memory emerged to be related to creativity also in children. Inhibition especially, which can be defined as the ability to suppress task-irrelevant response tendencies, seems to be able to predict creative performance during childhood, even when results are controlled for intelligence (Krumm et al. 2018). Moreover, in a recent work exploring domain-specific forms of creativity in primary school children, working memory (with the updating function) emerged as a central predictor of mathematical creativity (Stolte et al. 2020).

### 1.2. The Relationship between Teachers’ Beliefs about Creativity and Children’s Creative Potential

Similarly, self- and others’ beliefs about creativity have also emerged as central predictors of creative performance and achievement (Gralewski and Karwowski 2019). Differently from the research on executive functions, however, the role of others’ beliefs about creativity have been widely explored during childhood, especially in educational settings, as testified by recent meta-analyses on this topic (Gralewski and Karwowski 2019; Machts et al. 2016). The beliefs of the teachers about children’s creativity represent a contextual resource that can hamper or boost children’s creative performance. Teachers’ perceptions of their students’ abilities can influence both the teaching process (Hoge and Cudmore 1986) and the experience by the children of their education (Ready and Wright 2011). Usually, these perceptions are explored through the teachers’ ratings of students’ creativity. Several students’ characteristics are rated by the teachers, particularly creative abilities or personality traits, which are instead explored by creativity tests. The results of meta-analyses (e.g., Gralewski and Karwowski 2019) showed that the teachers’ ratings of their students’ creative abilities are indeed positively associated with the students’ actual creative performance, even if only at a low-to-moderate extent. Several studies highlighted the inaccuracy of teachers’ beliefs about the creativity of their students, showing low awareness about their students’ creative potential (Gralewski and Karwowski 2013; Scott 1999). Recently, instead of exploring the teacher’s ratings of students’ potential, using a social cognitive theory approach, Rubenstein et al. (2018) explored the teacher’s perceptions of the personal characteristics, behaviors, and environmental factors that can facilitate or inhibit creativity within a class. Consistent with the current definition of creativity (e.g., Corazza 2016; Plucker et al. 2004), this perspective not only underlines the importance of behavioral, personal, and environmental factors, but also empathizes the agentic action of the individual in the expression of the creative potential. The perceptions of the teachers about the factors influencing the educational environment for creativity could have an impact on the creative behavior of students, who however have a fundamental active role in the expression of their potential. Research showed that teachers can identify several macro-environmental factors that can inhibit their ability to accomplish their educational goals in creativity teaching, such as time constraints, required curriculum, or lack of administrator support (Rubenstein et al. 2018). Moreover, exploring implicit conceptions of creativity, Rubenstein et al. (2013) showed that teachers that believed that creativity had a high social value felt that most students could grow in their creativity, through the exploitation of their potential, and that they were capable of developing students’ creativity. However, it should be noted that the role of these implicit conceptions of teachers about the factors that influence creativity still needs to be fully understood, since, to the best of our knowledge, no study has ever explored their impact either on the actual children’s performance or on other factors defining children’s creative potential.

### 1.3. The Key Role of Trait Emotional Intelligence on Creative Potential

Embracing the view according to which creative potential is a confluent ensemble of various resources, the interactions between several components should be taken into account to characterize the creativity phenomenology in children. Among the main categories of resources included in the multivariate approach to the study of creativity (Lubart 1999; Sternberg and Lubart 1995; Sternberg and Lubart 1996), the present work takes the emotional resources as a central starting point for investigating the children’s creative potential. Emotional states and motivation have been indeed proposed to be the *conditio sine qua non* for the creative process to emerge (Agnoli and Corazza 2019). Motivation has been proposed in several theoretical and empirical approaches as the driving force for the creative process (Agnoli et al. 2018; Amabile 1993; Amabile et al. 2005). Moreover, the emotional states emerging during the process can be considered as fundamental resources, described by Lubart and Getz (1997) as emotional-based mechanisms leading to the generation of new ideas. Interestingly, the way individuals regulate, manage, and use the emotional resources that are invested and that emerge during the creative process is a major discriminant factor to define the outcome, in terms of success or failure, of this process.

With the present work, through two separate studies, we were interested in examining the interactive role of trait emotional intelligence (trait EI) over executive functions (Study 1) and teachers’ beliefs (Study 2) in the definition of children’s creative potential. Trait EI is defined as an ensemble of emotion-related dispositions and self-perceptions (Petrides et al. 2007), a collection of affect-related personality traits measurable with self-reports (Hughes and Evans 2018). Research showed that individuals varying in trait EI differ in the way they process, use, and manage emotional information (Petrides and Furnham 2003). Individual differences in trait EI emerged to be central for the positive adaptation within the classroom, assuming particular importance in the definition of children’s social–emotional competences and consequent adaptive behaviors with peers (Frederickson et al. 2012). Interestingly, this emotional personality trait emerged to be a discriminant variable to manage the emotional forces emerging during a creative process, and, in particular, during episodes of creative frustration (which are typical conditions during a creative process), and to use these forces to increase creative thinking performance (Agnoli et al. 2019b). Emotional intelligence indeed emerged to be a central element of the creative thinking process both in adults (Agnoli and Corazza 2019; Agnoli et al. 2019b; Lubart and Getz 1997; Sánchez-Ruiz et al. 2011; Zenasni and Lubart 2008) and in children (Hoffmann et al. 2020). It emerged to predict the beneficial or malevolent use of creative thinking (Harris et al. 2013) as well as the creative performance in the workplace by the mediating role of generosity and vigor (Carmeli et al. 2014) and has been presented as a central element for the expression of the individual creative potential (Agnoli 2021). Moreover, trait EI emerged recently to be an important variable that should be taken into account when exploring methods to increase children’s creative potential: trait EI interacted with the efficacy of a training intervention based on cognitive and metacognitive exercises aimed at increasing the creative potential of primary school children (Agnoli et al. 2022). It is finally worth highlighting that using a trait perspective in the exploration of emotional intelligence allows for taking into account both conative and affective resources influencing creativity. Trait EI can indeed be intended as a personality-conative resource that is at the basis of the perception, use, and management of the emotional resources.

### 1.4. The Present Work: Aims and Hypotheses

Emotions and the ability to manage and use emotional drivers during a creative process emerged therefore to be central variables to consider when exploring children’s creative potential. How the management and use of emotions (i.e., the level of emotional intelligence) can interact, by modulating the effect of other resources (i.e., cognitive and contextual) in defining creative potential is still an unexplored question, especially during development. In the present work we analyzed, in two separate studies, the interactive role of children’s trait emotional intelligence on the predictive effect of executive functions (Study 1) and of the teachers’ perception of the factors influencing creativity (Study 2) over children’s creative potential.

Across the two studies, our main hypotheses were related to the assumption of an interactive effect of emotional intelligence on the influence exerted by the two components, respectively, cognitive and environmental resources, on children’s creative potential. In particular, we expected that the positive effects of the cognitive resources (i.e., executive functions) on creative potential could emerge in children only when an adequate level of emotional intelligence was present. A low level of trait EI could indeed hamper the effect of the cognitive resources that children can use to express their creative potential, being the resources invested in the management of uncontrolled emotional states emerging during the execution of creative activities (see for instance the detrimental effect of high levels of stress on the influence of cognitive inhibition over creative performance shown by Duan et al. 2019). On the contrary, we hypothesized that a sufficient level of emotional intelligence could favor the control, inhibition, regulation, and use of the emotional forces emerging during a creative process, allowing the use of the executive functions in the expression of children’s creative potential.

Moreover, in accordance with previous research (Gralewski and Karwowski 2019), we hypothesized a positive effect of contextual resources associated with the teacher’s beliefs on creativity, especially in relation to their implicit conception of children’s creative potential and on their perceived self-efficacy to teach creativity. More importantly, we hypothesized that these teachers’ beliefs could be especially effective in children characterized by lower emotional intelligence resources. In such a case, it is indeed possible that children may need to rely on external resources to achieve in their creative activities. Put simply, we hypothesized that teacher’s conceptions about creativity could be helpful for the expression of the creative potential especially in children having low emotional resources in terms of trait EI. Finally, since gender differences have been extensively demonstrated in trait EI between girls and boys during childhood and adolescence (Agnoli et al. 2019a; Davis and Humphrey 2012; Mavroveli et al. 2008), we also took into consideration the role played by gender in these hypothesized effects. 

## 2. Study 1

### 2.1. Introduction

As mentioned before, the first study was devoted to the analysis of the interactive relationships between the cognitive and the emotional resources that define children’s creative potential. Based on results obtained in previous research on the relationship between specific executive functions and creative performance (Benedek et al. 2014; De Dreu et al. 2012; Edl et al. 2014; Krumm et al. 2018; Stolte et al. 2020), three executive functions, i.e., interference control, response inhibition, and working memory, were explored using experimental paradigms particularly suited to primary school children, as well as their interactions with trait EI in predicting children’s creative potential, as measured through the Evaluation of Potential Creativity (EPoC) instrument (Lubart et al. 2011), which is specifically developed for childhood.

### 2.2. Method

#### 2.2.1. Participants

A total of 187 children (females = 106) aged 8–11 years (*M* = 8.81, *SD* = .85) were recruited from two primary schools in Italy and participated in this study. Prior to testing, informed consents to children’s participation were obtained from parents or legal guardians. Children were free to participate in the study and were assured that they were free to withdraw from the study at any time without consequence. Approval from the Ethical Committee of the University of Bologna (Italy) was obtained before the study execution, which conformed to the Declaration of Helsinki.

#### 2.2.2. Procedure

Participants first completed a series of cognitive tasks examining three executive functions: the Simon task (Simon and Rudell 1967), the Flanker task (Eriksen and Eriksen 1974), and the n-back task (Kirchner 1958). Participants were tested collectively at a class level (3rd, 4th, 5th classes), and the order of tasks administration was randomly varied between classes. Children sat in front of a 14-in color monitor of a laptop at a viewing distance of approximately 40 cm. Stimulus presentation and response collection were controlled by the E-Prime (version 3) software system. To ensure that the children had time to become comfortable with the cognitive tasks, each task included a block of practice trials that was administered prior to data collection (see details below). Then, children’s creative potential was measured using the EPoC instrument (Lubart et al. 2011). Specifically, only the EPoC graphical tasks were used in the current study, in order to exclude possible effects of verbal proficiency in children with parents with a different mother tongue other than Italian. Trait EI was administered at the end of the session through the Trait Emotional Intelligence Questionnaire—Child Short Form (TEIQue-CSF; Mavroveli et al. 2008). The overall session lasted about 120 min. A 10-min break was administered after each executive functions task (i.e., 3 breaks), and before the TEIQue-CSF administration.

#### 2.2.3. Executive Functions

##### Interference Control: Simon Task

The stimuli and procedure were similar to those previously used in the literature with children of the same age (Iani et al. 2014). The stimuli were red or blue solid squares (3.6 × 3.6 cm), presented on a white background to the left or the right of a white fixation cross (.6 × .6 cm) with an eccentricity of 5.7 cm. Children responded to the stimuli by pressing the ‘ALT’ key (on the left side of the keyboard) or the ‘CTRL’ key (on the right side of the keyboard) with the left and right index finger, respectively. The keys were wrapped with the appropriate colored pieces of paper, and the keyboard was located centrally with respect to the body midline. Children were asked to respond as quickly and accurately as possible to the color of the stimulus by pressing the key of the same color, ignoring its position. The corresponding (C) condition occurs when the stimulus and response positions spatially correspond; otherwise, the noncorresponding (NC) condition occurs when they do not (e.g., Rubichi and Nicoletti 2006; Rubichi et al. 2006, for reviews). The experimenter read the instructions and ensured that they were understood by the children. Half of the participants answered to the red square with the left hand and to the blue square with the right hand, whereas the other half faced the inverse mapping rule. The task consisted of 128 trials divided into 4 blocks of 32 trials each, preceded by 20 practice trials. The children were given a break after each block. Each trial began with the presentation of a fixation cross, followed after 1000 ms by the stimulus, which persisted for 3000 msec or until a response was given. The trial ended if the participant did not respond within 3000 ms (no feedback was provided). The intertrial interval (ITI) was 2000 ms, during which the screen was blank. For each participant, correct responses that were 2.5 standard deviations above or below the mean response times (RTs in milliseconds) were excluded from the analyses. The Simon effect^2^, calculated by subtracting the mean RTs of corresponding (C) trials from those of noncorresponding (NC) trials, were analyzed. 

#### 2.2.4. Response Inhibition: Flanker Task

Again, the stimuli and procedure were similar to those previously used in the literature with children from the same age groups (Christ et al. 2011). Children were shown a stimulus comprised of a horizontal row of five yellow fish, and, on each trial, they were asked to respond as quickly as possible as to whether the center fish (target) was facing to the left or right. The target fish was always located in the same location (i.e., the center of the display) in each trial. Participants used both hands to respond and were asked to press the left button (‘ALT’ key) if the center fish was facing left, and the right button (‘CTRL’ key) if the center fish was facing right. Each individual fish stimulus subtended approximately 1 degree vertically and 1.9 degrees horizontally. The width of the full array of five fish was 10 degrees. Two types of trial were administered: compatible and incompatible. In compatible trials, the five fish in the stimulus array pointed in the same direction. In incompatible trials, the four distracting fish pointed in the opposite direction than the central target fish. In each trial, stimuli were presented until a response was given or until more than 3000 msec elapsed. After an intertrial interval (ITI) of 1500 msec, a new trial was presented. Children performed two practice blocks of 10 trials each. For trials in the first practice block, the center target fish was presented alone without any flanking fish. This allowed the children to become familiar with the task instructions and reinforced the fact that they should respond to the center target fish. In the second practice block, trials included a full array of five fish (i.e., a central target fish and four flanking distractor fish) and hence prepared the children for the upcoming experimental trials. After practice, children completed a total of 120 experimental trials (60 compatible and 60 incompatible trials) divided into 3 blocks of 40 trials each. Children were offered a break after each block. For each participant, correct responses that were 2.5 standard deviations above or below the mean were excluded from the analyses. The Flanker effect^3^ was computed by subtracting mean RTs on compatible (C) trials from those on noncompatible (NC) trials.

#### 2.2.5. Working Memory: N-Back Task

The version of the n-back task applied here followed the procedure described by Pelegrina et al. (2015), particularly suitable for use with children, and consisted of two levels: 1-back and 2-back. The items used were letters, in particular the following 20 consonants: B, C, D, F, G, H, J, K, L, M, N, P, Q, R, S, T, V, W, Y, Z. Stimuli were presented one by one in the center of the screen (font: Palatino Linotype, size: 30). On the 1-back task, the children had to compare the letter that was currently present on the screen to the one that was previously present on the screen; specifically, they were asked to press the ‘yes’ key (‘ALT’) only when the two letters were the same; otherwise, they were asked to press the ‘no’ key (‘CTRL’). The 2-back versions of the task were analogous, with the exception that on 2-back trials, children had to compare the letter that was currently present on the screen to the one shown two trials before. Each letter appeared on the screen for 500 msec, followed by a screen that remained blank for another 3000 msec. The children thus had 3500 msec from stimulus onset until the beginning of the subsequent trial to press the corresponding response key on the keyboard. Each level of the task (1-back and 2-back) started with instructions, followed by examples that contained a sequence of six letters with the corresponding correct responses. A practice block, which consisted of 10 trials, preceded the test block made up of 40 trials per level (30% “yes” trials). For each task level (1-back and 2-back), the signal-detection parameter d-prime (d′), estimated as *d′* = *Z_Hits_* − *Z_FalseAlarms_*, that reflects the sensitivity of the participants to discriminate items as previously presented (or not) *n* trials back, was analyzed.

#### 2.2.6. Creative Potential: Evaluation of Potential Creativity (EPoC)

Children’s creative abilities defining creative potential were measured through the Evaluation of Potential Creativity (EPoC; Lubart et al. 2011). The EPoC instrument is based on verbal and graphic tasks aimed at measuring explorative divergent thinking and integrative convergent thinking as two key modalities of creative cognition during development.

Both thinking modalities were assessed through two types of tasks, which include both abstract and concrete stimuli (Set A; see (Agnoli et al. 2022) for a similar procedure). Each child executed the EPoC tasks during a measurement session provided to the class group, after a warm-up trial that was presented in order to familiarize the children with the type of tasks used. Specifically, children were asked to perform (1) an abstract divergent–explorative task (producing as many alternative drawings as they could in 10 min starting from an abstract stimulus, e.g., a curved line); (2) an abstract convergent–integrative task (using at least four different abstract stimuli chosen among the eight presented, in order to produce, in 15 min, one original drawing); (3) concrete divergent–explorative and (4) convergent–integrative tasks, which were based on real-concrete stimuli (e.g., a banana) and were based on the same timing used for the abstract tasks. 

The scoring of children’s productions was performed by four raters trained in the use of the EPoC instrument. For both divergent–explorative tasks (abstract and concrete) a fluency score was computed, which consists in the total number of drawings produced by the child in the two tasks. The two fluency scores computed for the abstract and the concrete tasks, respectively, were then averaged in order to obtain a single score for the explorative divergent ability. For the convergent–integrative tasks originality scores were instead computed. Specifically, the children’s integrative ability was scored on a 1–7 score Likert scale, where 1 was assigned to a very poor, free of ideas drawing, and 7 to a drawing that contains high original ideas which integrate the elements in an innovative way. The raters were randomly assigned a series of drawings that they had to score in terms of originality. Similar to the fluency scores, the two originality scores for the integrative abstract and the concrete tasks were averaged into a single originality score.

#### 2.2.7. Emotional Intelligence: Trait Emotional Intelligence Questionnaire—Child Short Form (TEIQue-CSF)

Trait EI was measured through the Trait Emotional Intelligence Questionnaire—Child Short Form (TEIQue-CSF; Mavroveli et al. 2008). This self-report instrument is composed of 36 items answered on a 1–5 point Likert scale (for example: “When I feel sad, I try to do something to change my mood” or “It’s easy for me to understand how I feel”), whose scores range from 36 to 180 and it provides a global score of the child trait emotional intelligence. TEIQue-CSF, being a short, simplified version of the TEIQue scale developed for children, has been demonstrated to provide a reliable coverage of all aspects of children’s trait EI (in the age range 8–12) (Mavroveli et al. 2009; Mavroveli et al. 2008). Consistent with previous research (Mavroveli et al. 2009; Mavroveli et al. 2008), TEIQue-CSF showed good internal consistency (α = .81).

#### 2.2.8. Data Analyses

A series of Generalized Linear Models, one for each executive function (i.e., interference control, response inhibition, and working memory) in both creative performance indexes, explorative–divergent (fluency) and integrative–convergent (originality) thinking, were executed in SPSS 26. Separately for the two creativity indexes, the executive function index (i.e., Simon effect, Flanker effect, or D-prime scores) was entered as a continuous variable, gender (two levels: male, female) was entered in the models as a between-subjects factor, while trait EI was again included as a continuous variable. Main effects, two-way and three-way interactions between the previous variables were added to the models. Robust error estimation was used to control for the effect of outliers (Wu 2009).

### 2.3. Results

#### 2.3.1. Divergent Explorative Performance

**Figure 1:**
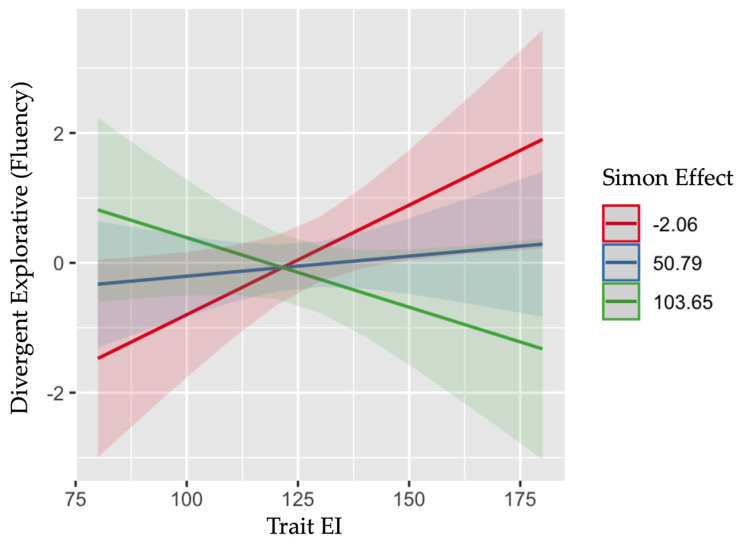
Interactive effect between trait emotional intelligence and the Simon effect level in predicting children’s divergent explorative ability (fluency). Low levels of the Simon effect correspond to a higher ability of interference control.

The analyses showed a significant main effect of the Simon effect, F(1, 160) = 5.875, *p* = .016 on the divergent explorative performance (fluency), which was further qualified by a significant interaction between the Simon effect and trait EI, F(1, 160) = 6.392, *p* = .012. As shown in Figure 1, divergent abilities increased with higher EI only in children exhibiting a smaller Simon effect, *b* = −.001, *t*(160) = −2.567, *p* = .011, 95% CI [−.001, .000]. No other main effects or interactions were significant (*ps* > .07). 

No significant main or interaction effect arises instead when considering both the Flanker effect (*ps* > .24) and the D-Prime computation from the 1-back task (*ps* > .09) and the 2-back task (*ps* > .38) on divergent explorative ability.

#### 2.3.2. Convergent Integrative Performance

**Figure 2:**
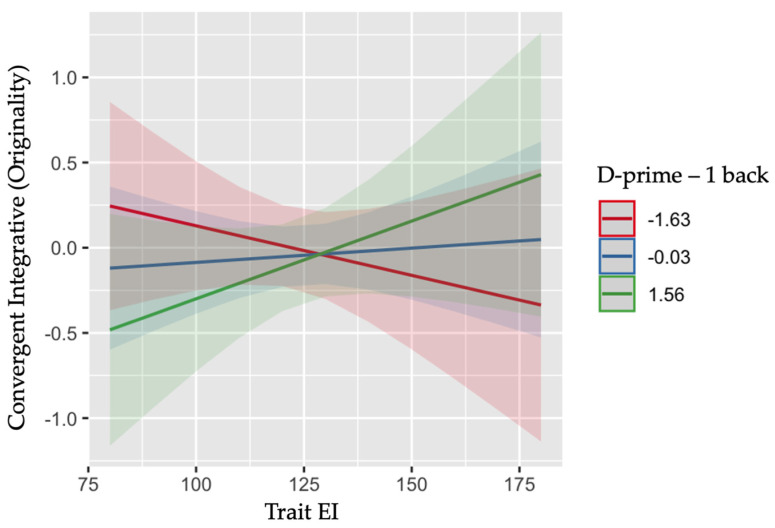
Interactive effect between trait emotional intelligence and n-back (1-back) performance in predicting children’s convergent integrative ability (originality). Larger D-prime values correspond to a higher working memory ability.

No significant main or interaction effect on originality emerged when considering the Simon effect (*ps* > .13) or the Flanker effect (*ps* > .35). On the contrary, the n-back performance emerged as a significant predictor of the convergent integrative ability, showing a significant main effect of D-prime in the 1-back task on originality, F(1, 133) = 5.881, *p* = .017. This main effect was further qualified by a significant interaction between D-prime and trait EI, F(1, 133) = 5.123, *p* = .025. As depicted in Figure 2, convergent abilities increase with higher EI only in children exhibiting a larger D-prime in 1-back task, *b* = .005, *t*(133) = 2.308, *p* = .023, 95% CI [.001, .009]. No other main or interaction effect was significant (*ps* > .70). Finally, no significant main or interaction effect arises instead when considering the D-Prime computation from the 2-back task (*ps* > .12).

### 2.4. Discussion

Overall, these results revealed a clear interactive effect of children’s emotional resources, measured in terms of their trait emotional intelligence, and cognitive resources, as measured through specific executive functions, regardless of gender, on children’s creative potential. Specifically, children who displayed higher ability to handle conflict resolution (i.e., smaller Simon effect) and to actively maintain and regulate, through a higher working memory ability a limited amount of task-relevant information (i.e., larger D-prime), show higher creative divergent–explorative and convergent–integrative thinking abilities, respectively, if these abilities combined with an adequate level of emotional intelligence. Overall, an adequate level of trait EI in children seems to play a key role in explaining the relationship between specific executive functions (i.e., interference control and WM) and their creative potential.

## 3. Study 2

### 3.1. Introduction

In the second study we focused on how emotional intelligence interacted with contextual factors influencing the creative climate in a classroom, i.e., the beliefs of the teachers, in predicting creative thinking. Put simply, can emotional intelligence emerge as an important variable when contextual variables are also considered for the expression of children’s creative potential? We again explored creative potential and trait emotional intelligence in primary school children using the same instruments adopted in Study 1. Gender was again considered as a possible variable interacting with trait EI in defining the hypothesized interactive dynamics.

### 3.2. Method

#### 3.2.1. Participants

A total of 448 children were involved in a larger study on the exploration and increase of children’s creative potential (see Agnoli et al. 2022). All participants were recruited from primary (third- to fifth-grade) state schools in three middle-sized cities in northern Italy. For the purposes of the present study, only participants who completed both the measure of creative potential and the measure of trait emotional intelligence and whose teachers completed the Teaching for Creativity Scale were considered for the analyses. Different research questions and different sets of data were therefore used in the current study in comparison to the already published research (Agnoli et al. 2022). Complete data were available for 344 pupils (176 females; age range = 8–11 years; mean age = 9.15 years; *SD* = 7.24 months).

A total of 19 classes for the three schools were involved in the study. Data of the teachers who were involved in the teaching activities of these classes were collected, taking into account their perception of the teaching for creativity. In total, 40 teachers (38 females; age range = 27–66 years; mean age = 48.37 years; *SD* = 1.48 years) were involved in the study. The over-representation of female teachers well reflects the actual proportions of teachers’ gender in Italian schools.

The present research conformed to the Declaration of Helsinki and was approved by the Bioethics committee of the University of Bologna. Parents gave their written consent for the study, and children and teachers were freely allowed to participate in or abstain at any time from the research.

#### 3.2.2. Instruments and Procedure

As mentioned above, the present study was part of a larger project on the study and the increase of children’s creative potential. During the project, a two-time (pre- and post-test) administration procedure was followed (see Agnoli et al. 2022). Here, only data pertaining to the first administration were taken into account (pre-test), in order to explore children’s creative potential without the effects of any planned intervention. Specifically, children’s creative potential and trait EI, as well as teachers’ perception of teaching for creativity were measured. 

#### 3.2.3. Creative Potential: Evaluation of Potential Creativity (EPoC)

Children’s creative potential was measured through the Evaluation of Potential Creativity (EPoC) using the same set of stimuli (set A) used in Study 1. Again, a fluency and an originality score were computed, which consisted of the average score between the scores obtained in the abstract and in the concrete tasks. The scoring of children’s productions was performed in this second study by six different raters trained in the use of the EPoC instrument. 

#### 3.2.4. Emotional Intelligence: TEIQue-CSF

Trait EI was measured through the Trait Emotional Intelligence Questionnaire—Child Short Form (TEIQue-CSF; Mavroveli et al. 2008). Consistently with Study 1, TEIQue-CSF showed a good internal consistency also in this second study (α = .73).

#### 3.2.5. Teachers’ Beliefs about the Teaching for Creativity: Teaching for Creativity Scale (TfCS)

We measured the teachers’ perceptions about the factors that can influence the teaching for creativity through the Teaching for Creativity Scale (TfCS; Rubenstein et al. 2013). TfCS measures four dimensions of the teacher’s beliefs about their efficacy in teaching creativity, about the importance of creativity in society, the supportive role of the school environment, and the students’ creative potential, namely: teacher self-efficacy (with items such as: “I am capable of fostering creative problem solving in my classroom”), environmental encouragement (with items such as: “It is a priority in my school to increase students’ inventiveness”), societal value (with items such as: “Innovative ideas can move society forward”), and student potential (with items such as: “All students can grow in their creative problem solving skills”). One score for each of these dimensions is provided by the scale. TfCS emerged to have a good internal reliability in the current study: teacher self-efficacy, α = .75, environmental encouragement, α = .70, societal value, α = .74, student potential, α = .78.

In order to match teachers’ beliefs with children’s scores, the average TfCS scores of the educators teaching in a class were associated with the scores in creative performance and trait EI of the children belonging to that specific class. Data emerged therefore to be organized at a class level. Moreover, since the educators taught a different number of hours per week in a class, before the computation of the average class scores, each TfCS score was weighed for the number of hours per week taught by the teacher in the class and a weighted mean was computed for each class, taking therefore into account the relative load of each teacher on the class beliefs. 

#### 3.2.6. Data Analysis

Given the multilevel nature of the data (students and teachers nested within classes and within schools), multilevel analyses were performed. Since the purpose of this study was to investigate the role of children’s trait EI and teachers’ beliefs about creativity on children’s creative potential, taking also into account the role of students’ gender, we focused only on the explanation of the within-class and within-school variability in the divergent and convergent performance. For this purpose, the variability in each creative performance that occurred between schools and classrooms was computed and controlled for through the use of Generalized Linear Mixed Models (GLMM) in SPSS 26.

Since the literature suggested that creative abilities show developmental variations in primary school children due to the effect of maturation (Claxton et al. 2005; Gralewski et al. 2016; Kim 2011) and age differences emerged in the grades here explored in divergent and convergent abilities as measured through the EPoC (Agnoli et al. 2022), the average EPoC scores for divergent and convergent tasks were centered around the mean performance in each grade (children’s age was classified into three age groups corresponding to the 3rd, 4th, and 5th grade—mean age was 8.29 years, 9.21 years, and 10.22 years, respectively). This computation allowed us to take into account the effect of the grade in the subsequent analyses.

Specifically, we were interested in exploring the role of teacher’s beliefs, children’s trait EI, and of their interaction in predicting children’s creative potential. Moreover, taking into account the gender difference in trait EI as emerged in past literature, we explored also whether gender, by interacting with trait EI and with teachers’ beliefs, can influence children’s creative potential. Thus, testing our hypotheses, two GLMM models (one for the explorative divergent performance and one for the integrative convergent performance) were executed with gender (two levels: male, female), trait EI (including children’s trait EI scores as a continuous variable), teacher self-efficacy, environmental encouragement, societal value, and student potential (all included as continuous variables) as predictors. Moreover, the two-way interaction between trait EI and each of the four teacher’s beliefs were included in the models, as well as the interaction between gender and trait EI, and the three-way interaction between gender, trait EI, and each of the four teachers’ beliefs.

### 3.3. Results

#### 3.3.1. Preliminary Analyses

Descriptive statistics of the divergent and convergent thinking performance, children’s trait EI, and teachers’ beliefs, as well as the correlations between these variables, are shown in Table 1. 

#### 3.3.2. Explorative Divergent Performance 

**Figure 3:**
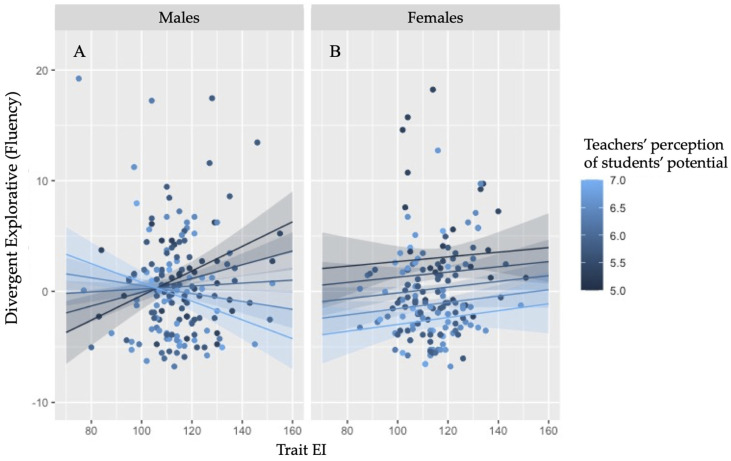
Interaction of trait emotional intelligence and teachers’ perception of students’ potential in predicting EPoC divergent thinking as a function of gender (males (**A**); females (**B**)).

The first GLM model performed on children’s explorative divergent performance highlighted a main effect of trait EI on divergent thinking as measured through EPoC, *b* = .574, *t*(312) = 2.236, *p* = .026, 95% *CI* [.069, 1.079]. This effect, in particular, highlighted that an increase of children’s emotional intelligence predicted an increase in their creative potential. Moreover, an interaction between trait EI and student potential in predicting divergent performance emerged, *b* = −.026, *t*(312) = −2.131, *p* = .034, 95% *CI* [−.051, −.002]. This interaction was further specified by a three-way interaction between trait EI, student potential and gender, *b* = .030, *t*(312) = 2.564, *p* = .011, 95% *CI* [.007, .053]. No other main effect of interaction effect emerged as significant in the analyses (*ps* > .120). As depicted in Figure 3, the three-way interaction seems to emerge as a consequence of the difference between boys and girls in the interaction effect between trait EI and student potential to predict divergent thinking performance. 

In males (Figure 3A) an evident reversal of the effect of teachers’ belief about students’ creative potential over divergent thinking performance emerged with the increase of trait EI. Indeed, when trait EI is low, high level of this belief predicted a higher divergent thinking performance in comparison to low levels. In contrast at high levels of trait EI, higher performance is associated with lower levels of this belief. In females (Figure 3B) no evident effect of the teachers’ belief or of trait EI seems to emerge. In order to further unravel this complex interaction effect, the effect of trait EI, of student potential, and of their interaction were explored in two separate GLM models in boys and girls. The model extracted from the boys’ data revealed a main effect of trait EI, *b* = .772, *t*(158) = 2.504, *p* = .013, 95% *CI* [.163, 1.382], an effect of student potential, *b* = 13.40, *t*(158) = 2.267, *p* = .025, 95% *CI* [1.724, 25.076], as well as, again, a two-way interaction between trait EI and student potential, *b* = −.126, *t*(158) = −2.481, *p* = .014, 95% *CI* [−.226, −.026] (Figure 3A). On the contrary, no main or interaction effect (*ps* > .295) emerged in females’ data (Figure 3B).

#### 3.3.3. Integrative Convergent Thinking Performance 

**Figure 4:**
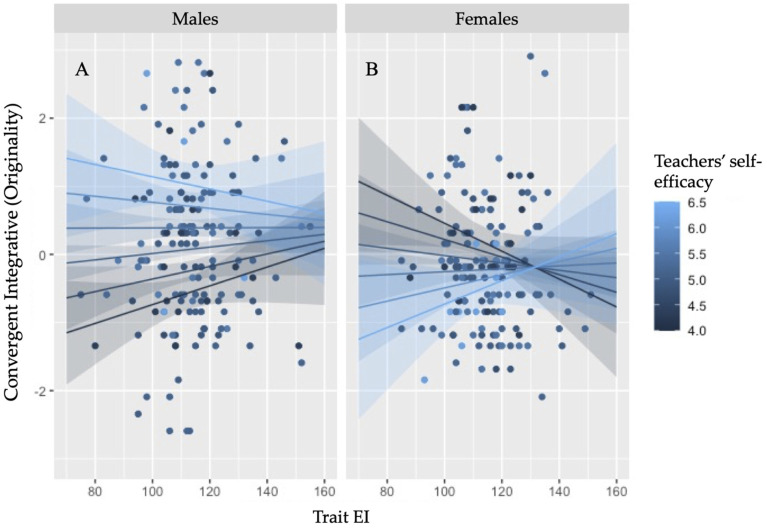
Interaction of trait emotional intelligence and teacher self-efficacy belief in predicting EPoC integrative thinking as a function of gender (males (**A**); females (**B**)).

The second GLM model performed on children’s integrative convergent thinking performance highlighted a three-way interaction between trait EI, teacher self-efficacy and gender, *b* = .008, *t*(312) = 2.535, *p* = .012, 95% *CI* [.002, .015]. No other main or interaction effect emerged between trait EI and the other teachers’ beliefs in association with gender (*ps* > .426). As depicted in Figure 4, trait EI influenced the effect of the teachers’ belief about their efficacy to teach creativity on student creative potential differently for males and females. Teachers’ belief emerged to influence boys’ creative potential only at low trait EI, while no effect of this belief emerged at high trait EI levels (Figure 4A). Specifically, results show that at low trait EI levels, the ability to integrate elements to generate original contents increased in males with the increase of teacher self-efficacy. On the contrary, in females at low trait EI levels student potential decreased with the increase of teacher self-efficacy, whereas this effect reversed at high trait EI levels. The increase of trait EI in females came with an increase of the positive effect of teachers’ self-efficacy on girls’ integrative ability (Figure 4B). 

### 3.4. Discussion

The results of the second study showed the important role of trait EI in specifying the effect of teachers’ beliefs about creativity on children’s creative potential. Trait EI indeed defined the direction of the effect of two teachers’ beliefs (i.e., student creative potential and teacher self-efficacy) in the prediction of divergent and integrative creative abilities of primary school children. Differently from Study 1, children’s gender also emerged as a central discriminant variable in this second study. Results revealed that the effect of divergent thinking on student potential was influenced by the teaches’ beliefs, but that the direction of this influence changed according to children’s trait EI. Specifically, at low trait EI levels, higher beliefs by the class teachers in the creative potential of students was associated with a higher divergent thinking performance; on the contrary, at high levels of children’s emotional intelligence, divergent thinking performance decreased with the increase of this teachers’ belief. It should be noted that this interaction effect emerged only in male students, whereas no effect emerged in girls, who do not appear to be influenced by trait EI and teachers’ beliefs in their divergent performance. 

Similarly, the integrative convergent performance emerged to be influenced by an interactive dynamic between children’s trait EI levels, teachers’ beliefs on their self-efficacy to teach creativity, and students’ gender. Specifically, teachers’ self-efficacy was effective in predicting boys’ integrative ability only at low trait EI levels. In girls, instead, the direction of teachers’ efficacy on the integrative ability reversed with the increase of trait EI: at low trait EI levels, higher levels of teachers’ self-efficacy were associated to a lower convergent thinking performance, whereas at high trait EI, a higher self-efficacy was associated with a higher convergent thinking performance. 

## 4. General Discussion

Starting from the notion that creativity stems from the dynamic interactions between different resources (Sternberg and Lubart 1991, 1996, 2022), the present work explored the interactive role of trait emotional intelligence over cognitive and contextual resources in defining the creative potential of primary school children. Specifically, in two different studies, we analyzed the modulatory role of trait EI over the effect exerted by executive functions and by teachers’ beliefs about creativity in the prediction of children’s exploratory–divergent and integrative–convergent thinking abilities. Therefore, our interest was mainly focused on understanding the role of the regulation, management, and use of emotions, as measured through trait EI, in the association of cognitive and contextual resources with children’s creative potential.

Consistent with the results emerging in adult (Beaty et al. 2014; Benedek and Fink 2019; Benedek et al. 2014; Edl et al. 2014; Zabelina et al. 2019) and developmental populations (Krumm et al. 2018; Sampedro and Pena 2019; Stolte et al. 2020), executive functions emerged as primary cognitive predictors of children’s creative potential. The ability to control interference, through a high conflict resolution, as measured by the Simon effect, was associated with a greater ability to produce alternative drawings starting from a graphical stimulus, i.e., to a higher ideational fluency. According to this result and in agreement with past findings (Edl et al. 2014), we can assume that a higher ability to solve the conflict in the selection of alternative responses can lead to a higher ability to effectively produce a higher number of alternative responses over time. More importantly for the purposes of the present study, this effect seems to emerge as a function of children’s trait EI level. The positive effect of conflict resolution over the divergent–explorative ability emerged with the increase of trait EI. The ability to solve cognitive conflicts was beneficial to the creative performance in terms of fluency only in children characterized by high levels of trait EI. We can therefore hypothesize that a sufficient self-perception to control, manage, and use emotions is needed to lead cognitive resources (in terms of control of interference) to affect children’s creative potential. We can assume that at low trait EI levels, children could be overwhelmed by the uncontrolled emotional reactions that arise during a creative process; it is thus possible that children’s cognitive resources might be engaged in the resolution of such emotional conflicts, more than on the resolution of the conflicts more strictly related to the creative task. On the contrary, at high trait EI levels, with a higher perceived control over the emotional forces acting during a creative process, the cognitive resources could be more engaged, or better channeled in the resolution of the cognitive conflicts related to the creative task. 

The very same effect emerged when working memory was explored as a predictor of the ability to integrate different elements to produce original products. The positive effect of working memory emerged in association with the increase of trait emotional intelligence. The ability to manipulate, add, or process different contents from the working system emerged therefore to be an important cognitive resource to generate integrated original products, confirming results from past research (De Dreu et al. 2012; Oberauer et al. 2008). However, our results highlighted that this ability emerged to be a positive predictor of creative potential especially when associated with a high perception by the children of their ability to regulate, manage, and use emotions. Its beneficial effect could instead be hampered in low trait EI children probably because their working memory could be engaged in the processing of uncontrolled emotional contents that emerged during the creative process. It is worth highlighting that this effect emerged when the 1-back results were analyzed and not in relation to the 2-back condition, where the higher difficulty of the task could probably have hidden the emergence of the trait EI effect. Two different executive functions emerged therefore as possible cognitive resources for the expression of diverse behavioral indexes of children’s creative potential: interference control in relation to the explorative divergent thinking ability and working memory as a predictor of the integrative convergent thinking performance. Moreover, and interestingly, the effects of these cognitive resources emerged to be modulated by the children’s trait EI level. Therefore, a sufficient level of emotional resources appears to be necessary for the expression of the beneficial effect of the cognitive resources over children’s creative potential, demonstrating an interacting relationship between the cognitive and the emotional components at the basis of the creativity phenomenology.

The children’s emotional resources emerged as a central moderator also in the analysis of the role of teacher’s conceptions about creativity on children’s creative potential. However, differently from the effect that emerged in the analysis performed on the role of executive functions, the potential positive effect of teachers’ beliefs seems generally to emerge when children were characterized by low emotional resources. In addition, when contextual factors influencing creative potential are taken into account, the typical difference between gender in emotional intelligence (Agnoli et al. 2019a; Davis and Humphrey 2012; Mavroveli et al. 2008) also emerged as a central variable in defining children’s creative potential. Specifically, the belief of the teachers about children’s creative potential had a beneficial effect on the divergent production of alternative drawings especially in boys characterized by low trait EI levels, whereas this beneficial effect disappeared (and seems to reverse) at high trait EI levels. On the contrary, in girls, who are usually characterized by a higher trait EI level, the beliefs of the educators teaching in their class did not influence their divergent explorative production. The conception of the teachers about students’ creative potential emerged to be beneficial especially in children characterized by low emotional resources, and specifically in boys characterized by low trait EI. Contextual resources seem to compensate in this case the lack of emotional resources: we might assume that a high confidence across the class teachers in student’s creative potential can encourage the expression of alternative responses especially in those children who are not able to manage the emotional reactions associated with a creative task. 

A similar effect emerged when considering the integrative convergent thinking ability in relation to the teachers’ belief about their efficacy to teach creativity. The effect of this belief emerged as significant in increasing children’s performance especially in low trait EI children, showing again a beneficial effect for male children characterized by lower emotional resources. However, an effect also emerged in girls, who showed an increase of their integrative convergent performance if teachers’ self-efficacy was associated with higher trait EI levels. Girls, who are usually characterized by higher trait EI levels than males, therefore emerged to benefit from teachers’ belief on their self-efficacy if this belief is combined with high emotional resources. In sum, teachers’ belief about their efficacy to teach creativity emerged to be beneficial for the generation of original products in boys characterized by low emotional resources, whereas it emerged to increase female creative performance at high trait EI levels. This difference between boys and girls on the modulatory effect of trait EI over the effect of teachers’ implicit conceptions on children’s creative thinking is worth to be further explored. By designing specific experimental approaches (or training interventions) based on the manipulation of the levels of trait EI in boys and girls, for instance, future research could try to understand and unravel whether this complex interaction effect depends on the typical gender difference in trait EI usually emerging during childhood (Agnoli et al. 2019a; Davis and Humphrey 2012; Mavroveli et al. 2008). 

The effects emerging from the analysis of the interaction between trait EI and a class contextual resource such as the teachers’ conceptions about creativity highlighted that different teachers’ beliefs can impact on children’s creative potential, showing evident differences between boys and girls. Boys, who are usually characterized by lower trait EI levels than girls (Agnoli et al. 2019a; Davis and Humphrey 2012; Mavroveli et al. 2008), emerged to benefit from teachers’ beliefs about students’ potential and about the efficacy to teach creativity especially when they were characterized by low emotional resources; these beliefs seem therefore to help the expression of their creative potential even in the absence of an important creative resources such as the control and management of their emotional reactions. We can instead assume that girls, who might be characterized by a sufficient level of emotional resources, do not usually benefit from teachers’ beliefs for the expression of their creative potential, unless these external resources resonate with a high level of management of emotions, i.e., with higher trait EI levels.

These results suggest that when developing programs or training interventions for the management and increase of creative potential, it would be particularly important to take into account both cognitive and emotional resources in the students as well as the teachers’ perception of the resources that can be used to teach creativity. Therefore, we suggest that, starting from successful trainings of creative potential (see Scott et al. 2004a, 2004b) and trait emotional intelligence (see Nelis et al. 2009), integrated interventions targeting children’s cognitive and emotional aspects of creative potential and teachers’ perception of creativity should be developed, thus involving both students and teachers.

### Limitations and Future Directions

It is important to highlight that if we consider the creative potential as constituted by a multitude of resources, these resources should be explored at the most complex level as possible to understand their mutual interactive dynamics. The present work however explored in two separate studies, instead of in a single study, how trait emotional intelligence (i.e., emotional resources) modulate cognitive and contextual resources, respectively, in predicting child’s creative potential. As a consequence, separate conclusions can be drawn on the diverse interactive role of trait EI over the two forms of resources for the expression of children’s creative potential. We therefore suggest that, on the basis of the effects emerged in the present work, future studies should explore the relative interactive role of trait EI over the executive functions and teachers’ beliefs in a single multiple model, which should highlight the weighted role of these different resources in the expression of children’s creative potential. Moreover, it is worth remembering that the executive functions as well as the teacher’s beliefs about creativity here explored are only some simplified proxies of the ensemble of cognitive and contextual resources that can affect the child’s creative potential. Further studies are therefore needed, exploring and integrating different forms of cognitive resources (further executive functions, attentive processing, memory structuration, intelligence, etc.) and contextual resources (socio-economic status, parents’ beliefs about creativity, teachers’ beliefs on the potential of single students, knowledge domain constraints, etc.) in interaction with children’s emotional intelligence. Likewise, others personality traits (e.g., openness, extroversion, etc.) besides trait EI could modulate the relationship between cognitive and contextual resources on children’s creative potential. Finally, at a more methodological viewpoint, in applying the EPoC instrument, we specifically investigated its graphical dimension; however, we call for future studies also exploring its verbal dimension.

## 5. Conclusions

Trait EI emerged from the present work as a discriminant variable to understand the effect of executive functions and teachers’ beliefs about creativity over children’s creative potential. This work highlights that the level of children’s emotional intelligence should be taken into account in the exploration of and the planning of interventions on children’s creative potential, since the emotional forces acting during a creative activity could potentially influence the effect of any other factor affecting the creative phenomenology. Individual differences in the self-perception of the regulation, management, and use of emotions can indeed explain potential differences in the diverse outcomes of a creative process. Moreover, our results suggest that differences in emotional intelligence might help explain eventual gender differences emerging at younger ages in the expression of creative potential (Ivcevic et al. 2022). With this work, a path is traced to include trait emotional intelligence within the explicatory variables of the creative potential in primary school children.

## Figures and Tables

**Table 1:** Descriptive statistics and correlations (Pearson R and number of participants, in brackets, are shown) between the average performance in the EPoC divergent and convergent tasks, children’s trait EI, and teacher’s beliefs.

		Mean	SD	Min	Max	1	2	3	4	5	6	7
1	Divergent Thinking	8.45	4.55	1.50	28	-						
2	Convergent Thinking	4.01	1.09	1	7	.089 (344)	-					
3	Children’s Trait EI	114.41	13.93	47	180	.060 (344)	−.005 (341)	-				
4	Teacher’s self-efficacy	5.11	.41	4.35	6.26	−.09 (328)	.037 (328)	−.039 (325)	-			
5	Environmental Encouragement	4.95	.63	3.43	5.84	.146 ** (328)	−.081 (328)	.131 * (325)	.130 (40)	-		
6	Societal Value	5.59	.41	4.90	6.30	−.273 ** (328)	−.086 (328)	−.137 * (325)	.354 * (40)	−.202 (40)	-	
7	Student Potential	6.07	.48	5.34	6.91	−.210 ** (328)	.117 * (328)	−.124 * (325)	.539 ** (40)	−.029 (40)	.469 ** (40)	-

*Notes*. * *p* < .05, ** *p* < .01. The number of participants (in brackets) refers to the two samples of participants tested in the study, i.e., children and teachers.

## Data Availability

The data presented in this study are available on request from the corresponding author.

## Notes

1 Conative resources typically include motivational aspects as well as personality-based characteristics (see Barbot et al. 2015).

2 As expected, the main effect of trial type was significant (*F*_1,169_ =156.73; *p* < .001, ηp^2^ = .48), with slower average reaction times following noncorresponding responses (*M* = 751.15 ms) in comparison to the reaction times following corresponding responses (*M* = 699.74 ms).

3 As expected, the main effect of trial type was significant (*F*_1,171_ =8.54; *p* < .001, ηp^2^ = .32), with slower average reaction times following noncompatible responses (*M* = 825.84 ms) than following compatible responses (*M* = 776.58 ms).
